# The relationship between nurse managers' empowering behaviors and nurses' work engagement: a latent profile analysis

**DOI:** 10.3389/fpubh.2026.1811404

**Published:** 2026-05-05

**Authors:** Man Li, Xinhua Xie, Hui Wang, Lei Ma, Jingfang Zhao

**Affiliations:** Department of Gastroenterology, Shandong Provincial Hospital Affiliated to Shandong First Medical University, Jinan, China

**Keywords:** general hospital, latent profile analysis, nurse managers' empowering behaviors, survey study, work engagement

## Abstract

**Aims:**

To explore the latent profiles of nurse managers' empowering behaviors in Chinese tertiary general hospitals and to analyze differences in nurse work engagement across these profiles, providing evidence for targeted nursing management interventions.

**Design:**

A cross-sectional study.

**Methods:**

Participants were 447 clinical nurses recruited from three hospitals in Shandong Province, China. Data were collected using a demographic questionnaire, the Nurse Managers' Empowering Behavior Scale, and the Utrecht Work Engagement Scale (UWES-9). Latent profile analysis (LPA) was used to identify distinct profiles of empowering behaviors. One-way ANOVA was applied to compare work engagement scores across profiles.

**Results:**

Nurse managers' empowering behaviors exhibited significant heterogeneity and were classified into four latent profiles: Detached-Control, Task-Driven, Utilitarian-Empowering, and Harmonious-Empowering. Significant differences in nurses' work engagement were observed across these profiles (*p* < 0.05). Compared with the other three profiles, nurses in the Harmonious-Empowering profile reported significantly higher total work engagement and higher scores across all dimensions (*p* < 0.05).

**Conclusion:**

Nurse managers' empowering behaviors are not homogeneous but exhibit distinct subgroup patterns. Comprehensive empowering behavior is a key managerial factor in enhancing nurses' work engagement. Nursing managers should strive to build an integrated empowering model that encompasses emotional support, delegation of authority, and competence development, thereby effectively stimulating nurses' work engagement and improving the quality of nursing care.

**Impact:**

Using latent profile analysis, this study is the first to systematically identify four distinct empowering behavior profiles of nurse managers in China. Findings indicate that instrumental support alone is insufficient to foster deep work engagement. The Harmonious-Empowering leadership style—integrating high respect, genuine care, and substantial autonomy—proves most effective in enhancing both work engagement and professional wellbeing. This evidence supports a shift in nursing leadership development from skill-based training toward an integrated humanistic model.

## 1 Introduction

Nurses play a vital role in the healthcare system. As the first point of contact for patients, they engage in ongoing communication with those under their care. Within clinical settings, nurses serve as an indispensable cornerstone of hospitals, dedicating themselves to saving lives and delivering essential nursing services (1). However, there is a vast worldwide shortage of nearly six million nurses (2). In this context, in addition to retaining existing nurses and accelerating the training of new ones, it is critical to enhance the work engagement of the current nursing workforce to alleviate the shortage of nursing staff (2). Work engagement, defined as “a positive, fulfilling work-related state of mind,” is characterized within the nursing profession by three core dimensions: vigor, dedication, and absorption (3). Vigor refers to the experience of high energy, mental resilience, enthusiasm, and persistence while working, especially when facing difficulties (4). Dedication entails a strong sense of involvement in one's work, accompanied by feelings of significance, challenge, pride, and inspiration (5). Absorption is characterized by being fully immersed in one's tasks, often leading to a state in which time seems to pass quickly (6, 7). Research indicates (8) that nurses with high work engagement typically demonstrate greater vitality, energy, and focus in their roles. They also exhibit stronger organizational commitment (9), enhanced work ability (10), higher retention intention (11), and improved work quality (12). Conversely, low work engagement can increase work pressure, heighten the risk of burnout, and compromise patient safety (6, 13). This not only undermines the stability of the nursing workforce but also negatively impacts the quality of clinical care.

As leaders of nursing teams, nurse managers' behaviors significantly influence nurses' work engagement. They play a crucial role in fostering a positive work environment through supportive leadership. Supportive empowering leadership behaviors are specifically manifested in: assisting in overcoming work difficulties, enhancing work meaning, recognizing work achievements, respecting staff, and granting autonomy (14, 15). “Assisting in overcoming work difficulties” is related to supporting staff nurses in overcoming their work-related problems by themselves; “recognizing work achievements” refers to evaluating their work and encouraging the use of results; “enhancing work meaning” refers to helping staff nurses find their job meaningful and helping them understand the purpose of their work; “respecting staff” is associated with interactions with staff nurses and respecting them as staff members; “granting autonomy” implies placing trust in the nurses, leaving the work up to staff nurses, encouraging self-determination and autonomy, and respecting their opinions. Such behaviors can provide nurses with crucial job resources and psychological support (14). Previous studies (16–18) have demonstrated a positive correlation between empowering leadership and employee engagement. Empowering leadership delegates authority and grants employees moderate autonomy, fostering a sense of responsibility and control that stimulates intrinsic motivation and enhances work engagement (19, 20). When nurse managers emphasize support, trust, empowerment, and autonomy, nurses develop a stronger sense of competence and meaningfulness, which enhances their psychological empowerment and ultimately leads to greater work engagement (16). However, this evidence comes primarily from research involving corporate employees, and existing studies in nursing have focused mainly on the effects of transformational (21), ethical (22), and authentic leadership (23) on nurses' professional outcomes, with empowering leadership receiving comparatively less attention.

Furthermore, previous research on empowering leadership has predominantly adopted a variable-centered approach, which limits the ability to capture individual differences within groups. Latent profile analysis (LPA), as a person-centered method, can reveal hidden subgroup structures in the data, identify distinct latent profiles or subpopulations, and contribute to a better understanding of heterogeneity within the sample (24). Latent profile analysis (LPA) has recently been applied to leadership research, with studies focusing on transformational leadership (25) and toxic leadership (26). The application of LPA in this area has been shown to yield sound scientific validity and effectiveness. This study aims to use Latent Profile Analysis to examine the latent profiles of clinical nurses' perceptions of nurse managers' empowering behaviors and their association with work engagement, thereby providing evidence for designing more targeted human resource management measures.

The research questions are as follows: (1) What are the latent profiles of clinical nurses' perceptions of nurse managers' empowering behaviors? (2) What is the association between different profiles of nurse managers' empowering behaviors and nurses' work engagement?

## 2 Methods

### 2.1 Design and participants

This study employed a cross-sectional design. A total of 447 nurses were recruited via convenience sampling from 3 tertiary hospitals in Shandong Province, China. The inclusion criteria were as follows: (1) being a frontline clinical nurse holding a valid Chinese nursing license; (2) having at least 1 year of clinical work experience; and (3) providing informed consent voluntarily. Nurses were excluded if they were (1) interns, visiting nurses, or those in standardized residency training; or (2) on leave during the survey period (e.g., sick leave or maternity leave).

### 2.2 Sample size

Based on existing scholarly evidence, the minimum sample size for this study was calculated to be at least 5–10 times the number of independent variables in the questionnaire. Given the inclusion of 19 independent variables and a potential 10%−15% invalid response rate, the theoretical sample size was calculated to be 105–219 nurses; however, the final sample size was set at 447 nurses.

### 2.3 Measures

#### 2.3.1 Information in general

The collected demographic and work-related characteristics included gender, age, department, marital status, profile of employment, years of work experience, educational level, professional title, whether they served as a clinical instructor, and whether they were a specialty-certified nurse.

#### 2.3.2 Nurse managers' empowering behavioral scale

The nurse managers' empowering behavioral scale was developed by Sasaki (14) and later translated and adapted into Chinese by Wen Juan (15). It is used to assess the level of empowering behavior exhibited by nurse managers as perceived by clinical nurses. The scale comprises five dimensions: assisting in overcoming work difficulties, enhancing work meaning, recognizing work achievements, respecting staff, and granting autonomy, with a total of 47 items. Responses are rated on a five-point Likert scale ranging from one (“strongly disagree”) to five (“strongly agree”). The total possible score is 235, with higher scores indicating a greater degree of empowering behavior demonstrated by the nurse managers. The overall Cronbach's α coefficient of the scale was 0.957, with coefficients for each dimension of 0.971, 0.946, 0.974, 0.964, and 0.970, respectively (15); in this study, the overall coefficient was 0.995, and the dimensional coefficients were 0.975, 0.981, 0.980, 0.984, and 0.971, respectively.

#### 2.3.3 Work engagement scale

Work engagement was measured using the 9-item Utrecht Work Engagement Scale (UWES-9), originally developed and shortened by Schaufeli (27), and subsequently translated and adapted into Chinese by Li Fuye (28). The scale consists of three dimensions: vigor, dedication, and absorption, encompassing a total of nine items. Responses are rated on a seven-point Likert scale ranging from 0 (“never”) to 6 (“every day”). The total score ranges from 0 to 54, with higher scores indicating a greater level of work engagement. The Cronbach's α coefficient of this scale was 0.930 in previous studies, with the subscale coefficients for vigor, dedication, and absorption being 0.780, 0.800, and 0.810, respectively (28); in this study, the overall coefficient was 0.938, and the subscale coefficients were 0.738, 0.883, and 0.895, respectively.

### 2.4 Data collection

The questionnaire was developed using the online platform Questionnaire Star, and the survey was conducted anonymously. A uniform instruction was provided at the beginning, outlining the study's purpose, significance, and completion guidelines. To ensure data quality, each IP address was restricted to a single submission, all items were set as mandatory, and participants were required to provide informed consent before proceeding with the anonymous survey. Finally, all responses were reviewed by two researchers. We distributed 481 questionnaires across three tertiary hospitals in Shandong Province, China, and received 447 valid responses, representing an effective response rate of 93.0%.

### 2.5 Ethical consideration

This study was approved by the Institutional Ethics Committee prior to implementation (Approval No. SWYX: NO.2025-428). All participants provided signed informed consent before data collection commenced. Data were collected anonymously, and all personal information was kept strictly confidential.

### 2.6 Data analysis

Data analysis was performed using Mplus 8.3 and SPSS 25.0 software. Normally distributed continuous variables were presented as mean ± standard deviation, while categorical variables were expressed as frequency and percentage. Inter-group comparisons were conducted using analysis of variance (ANOVA) or the chi-square (χ^2^) test. For *post-hoc* pairwise comparisons following ANOVA, the Least Significant Difference (LSD) test was applied when homogeneity of variances was assumed; otherwise, the Games-Howell test was used (29). A significance level of *p* < 0.05 was considered statistically significant.

Using Mplus version 8.3, a latent profile analysis (LPA) was conducted with the robust maximum likelihood estimator (MLR). By providing robust standard errors, the MLR estimator enhances classification reliability and enables an accurate exploration of the latent profiles of nurse managers' empowering behaviors (30). The LPA was conducted using the means of the five dimensions of nurse managers' empowering behaviors as the explicit variables. The number of potential profiles ranged from 1 to 5. The optimal number of profiles was determined by evaluating several model fit indices, including the Akaike information criterion (AIC), Bayesian information criterion (BIC), adjusted BIC (aBIC), entropy index, Lo-Mendell-Rubin test (LMR), and the Bootstrap Likelihood Ratio Test (BLRT). Lower values of AIC, BIC, and aBIC indicate a better fit, while significant results (*p* < 0.05) from LMR and BLRT suggest that the k-class model outperformed the k-1 class model. Classification accuracy was assessed using the entropy index, with values above 0.8 indicating high classification accuracy (24, 31–33). Based on these fit indices, the optimal latent profile model for nurse managers' empowering behaviors was determined.

## 3 Results

### 3.1 Demographic characteristics of clinical nurses

A total of 447 clinical nurses were included in the final analysis. Among them, 34 (7.6%) were male and 413 (92.4%) were female. The age of participants ranged from 22 to 54 years, with a mean (± standard deviation) of 32.93 ± 6.66 years. Regarding educational background, the majority held a bachelor's degree (*n* = 392, 87.5%), followed by 36 (8.0%) with a master's degree. In terms of departmental distribution, 175 nurses (39.1%) worked in internal medicine-related units, 88 (19.6%) in surgical departments, and 136 (30.4%) in emergency/ICU/operating room settings. Additionally, 57 nurses (12.7%) served as clinical instructors. Detailed demographic information is presented in Table 1.

**Table 1:** Univariate analysis of nurse demographics across different profiles of nurse managers' empowering behaviors (*N* = 447).

Variables	Number of person	Percent (%)	Detached-control profile	Task-driven profile	Utilitarian-empowering profile	Harmonious-empowering profile	*t*/*x*^2^	*P*
Gender								
Male	34	7.6	0 (0%)	3 (8.8%)	17 (50%)	14 (41.2%)	4.547	0.208
Female	413	92.4	16 (3.9%)	82 (19.9%)	188 (45.5%)	127 (30.8%)		
Department								
Internal medicine	175	39.1	11 (6.3%)	41 (23.4%)	81 (46.3%)	42 (24.0%)	22.517	0.095
Surgery	88	19.7	3 (3.4%)	10 (11.4%)	38 (43.2%)	37 (42%)		
Emergency	46	10.3	0 (0.0%)	8 (17.4%)	26 (56.5%)	12 (26.1%)		
Operating	27	6.0	0 (0.0%)	4 (14.8%)	14 (51.9%)	9 (33.3%)		
ICU	63	14.1	1 (1.6%)	11 (17.5%)	26 (41.3%)	25 (39.7%)		
Others	48	10.7	1 (2.1%)	11 (22.9%)	20 (41.7%)	16 (33.3%)		
Years of working (years)								
≤ 30	82	35.6	1 (1.2%)	14 (17.1%)	47 (57.3%)	20 (24.4%)	13.010	0.162
31–40	93	20.8	3 (3.2%)	17 (18.3%)	47 (50.5%)	26 (28.0%)		
40–50	223	49.8	8 (3.6%)	46 (20.6%)	94 (42.2%)	75 (33.6%)		
≥50	49	11.0	4 (8.2%)	8 (16.3%)	17 (34.7%)	20 (40.8%)		
Marital status								
Single	159	35.6	5 (3.1%)	30 (18.9%)	87 (54.7%)	37 (23.3%)	10.857	0.093
Married	285	63.8	11 (3.9%)	55 (19.3%)	116 (40.7%)	103 (36.1%)		
Divorced	3	0.7	0 (0.0%)	0 (0.0%)	2 (66.7%)	1 (33.3%)		
Employment form								
Contract system	360	80.5	11 (3.1%)	71 (19.7%)	170 (47.2%)	108 (30.0%)	94.948	0.624
Formal preparation	45	10.1	1 (2.2%)	10 (22.2%)	16 (35.6%)	18 (40.0%)		
Others	42	9.4	4 (9.5%)	4 (9.5%)	19 (45.3%)	15 (35.7%)		
Years of working (years)								
≤ 5	155	34.7	4 (2.6%)	26 (16.8%)	88 (56.8%)	37 (23.9%)	16.696	0.054
6–10	74	16.6	2 (2.7%)	19 (25.7%)	26 (35.1%)	27 (36.5%)		
11–20	188	42.1	9 (4.8%)	35 (18.6%)	81 (43.1%)	63 (33.5%)		
>20	30	6.7	1 (3.3%)	5 (16.7%)	10 (33.3%)	14 (46.7%)		
Education level								
Associate's degree	19	4.3	0 (0.0%)	6 (31.6%)	9 (47.4%)	4 (21.1%)	11.175	0.083
Undergraduate	392	87.7	12 (3.1%)	75 (19.1%)	177 (45.2%)	128 (32.7%)		
Postgraduate	36	8.1	4 (11.1%)	4 (11.1%)	19 (52.8%)	9 (25.0%)		
Professional title								
Nurse	92	20.6	2 (2.2%)	18 (19.6%)	47 (51.1%)	25 (27.2%)	18.914	0.026
Senior nurse	118	26.4	1 (0.8%)	23 (19.5%)	64 (54.2%)	30 (25.4%)		
Nurse-in-charge	233	52.1	12 (5.2%)	44 (18.9%)	93 (39.9%)	84 (36.1%)		
15.5-7.4,-13.5175.3mmAssociate chief nurse	4	0.9	1 (25.0%)	0 (0.0%)	1 (25.0%)	2 (50.0%)		
Clinical instructor?								
No	390	87.2	13 (3.3%)	77 (19.7%)	183 (46.9%)	117 (30.0%)	4.432	0.218
Yes	57	12.8	3 (5.3%)	8 (14.0%)	22 (38.6%)	24 (42.1%)		
Specialist nurse?								
No	351	78.5	9 (2.6%)	70 (19.9%)	166 (47.3%)	106 (30.2%)	14.291	0.112
Yes	96	21.5	7 (7.3%)	15 (15.6%)	39 (40.6%)	35 (36.5%)		

### 3.2 Descriptive results of nurse managers' empowering behaviors and nurses' work engagement

Work Engagement score was 44.75 ± 12.32 points; the nurse managers' empowerment score was 188.33 ± 38.54 points. Specific scores are shown in Table 2.

**Table 2:** Scores on nurses' work engagement and nurse managers' empowering behaviors (*N* = 447).

Variables	Mean	SD
Work engagement	44.75	12.32
Vigor	21.00	14.88
Dedication	21.00	14.80
Absorption	21.00	15.07
Nurse managers' empowering behaviors	188.33	38.54
Assisting in overcoming work difficulties	40.32	8.25
Enhancing work meaning	40.15	8.25
Recognizing work achievements	40.14	8.22
Respecting staff	35.76	8.01
Granting autonomy	31.96	6.54

### 3.3 Correlation of research variables

The results indicated that nurse managers' empowering behaviors were positively correlated with work engagement (*P* < 0.001), as shown in Table 3.

**Table 3:** Correlations between key study variables (*n* = 447).

Variables	1	2	3	4	5	6	7	8	9	10
Work engagement	1	–	–	–	–	–	–	–	–	–
Vigor	0.951^**^	1	–	–	–	–	–	–	–	–
Dedication	0.939^**^	0.850^**^	1	–	–	–	–	–	–	–
Absorption	0.934^**^	0.845^**^	0.792^**^	1	–	–	–	–	–	–
Nurse managers' empowering behaviors	0.493^**^	0.466^**^	0.487^**^	0.438^**^	1	–	–	–	–	–
Assisting in overcoming work difficulties	0.490^**^	0.460^**^	0.482^**^	0.440^**^	0.983^**^	1	–	–	–	–
Enhancing work meantime	0.492^**^	0.464^**^	0.488^**^	0.435^**^	0.993^**^	0.974^**^	1	–	–	–
Recognizing work achievements	0.488^**^	0.463^**^	0.485^**^	0.427^**^	0.988^**^	0.961^**^	0.977^**^	1	–	–
Respecting staff	0.480^**^	0.456^**^	0.473^**^	0.424^**^	0.984^**^	0.957^**^	0.971^**^	0.960^**^	1	–
Granting Autonomy	0.485^**^	0.456^**^	0.475^**^	0.436^**^	0.987^**^	0.959^**^	0.976^**^	0.977^**^	0.964^**^	1

^**^p < 0.001.

### 3.4 Latent profile analysis of nurse managers' empowering behaviors

**Figure 1:**
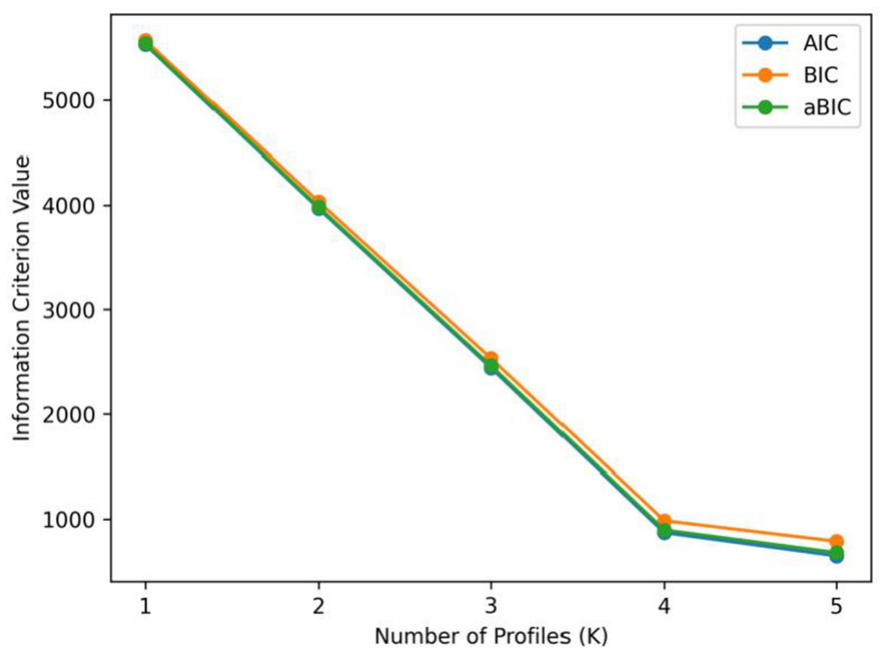
Elbow plot for model selection.

Using the mean scores of the five dimensions of the Nurse Managers' Empowering Behavior Scale as manifest indicators, latent profile models with one to five profiles were fitted. The model fit indices are presented in Table 4. As the number of profiles increased, the values of AIC, BIC, and aBIC progressively decreased, and the entropy values for all models exceeded 0.90. Notably, the information criteria decreased rapidly until the four-profile model, after which the decline slowed when the number of profiles increased from four to five (Figure 1). The Lo–Mendell–Rubin test (LMRT) yielded significant *p*-values for the one- and four-profile models, but not for the two- or five-profile models. Among all solutions, the four-profile model achieved the highest entropy value of 0.993. The average latent class assignment probabilities for the four profiles were 4% (*n* = 18), 19% (*n* = 85), 45% (*n* = 201), and 32% (*n* = 143), indicating reliable classification and meeting the criteria for an appropriate latent profile model. Therefore, based on a comprehensive evaluation of the fit indices and practical interpretability, the four-profile model was selected as the optimal solution in this study.

**Table 4:** Fit indices for the latent profiles of nurse managers' empowering behaviors.

Profile	AIC	BIC	aBIC	LMRT(p)	BLRT(p)	Entropy
1	5530.223	5571.248	5539.515	/	/	/
2	3966.931	4032.572	3981.794	0.0003	0.0000	0.966
3	2442.054	2532.310	2462.491	0.2924	0.0000	0.980
4	870.816	985.688	896.827	0.0022	0.0000	0.993
5	648.739	788.226	680.324	0.2989	0.0000	0.982

**Figure 2:**
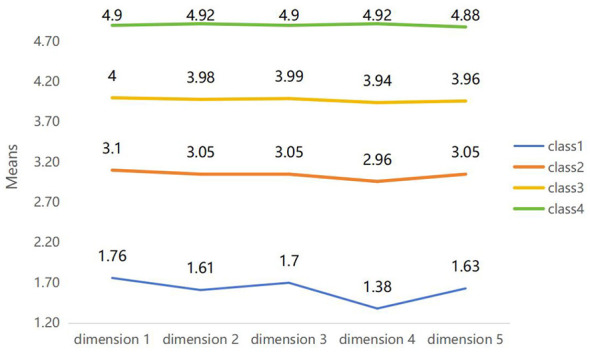
Latent profile model of nurse managers' empowering behaviors.

The characteristics of the four latent profiles of nurse managers' empowering behaviors were analyzed and named based on the mean scores across the five dimensions (Table 5, Figure 2). Profile 1 was named the Detached-Control profile, as mean scores across all dimensions were at a relatively low level. Profile 2 was classified as the Task-Driven profile. All dimensions received moderate scores ranging from 2.96 to 3.10. Although the pattern was relatively balanced across dimensions, the “respecting staff” dimension scored lower. Profile 3 was termed the Utilitarian-Empowering profile. Its mean scores across dimensions were higher than those of Profile 2 but lower than Profile 4, with a notably lower score in the “respecting staff” dimension. Profile 4 was identified as the Harmonious-Empowering profile, demonstrating relatively higher scores across all dimensions, especially the highest in “respecting staff. ”

**Table 5:** Comparison of mean scores across dimensions by latent profile of nurse managers' empowering behaviors ($\bar{x}$ ± s).

Class	Assisting in overcoming work difficulties	Enhancing work meaning	Recognizing work achievements	Respecting staff	Granting autonomy
Detached-Control profile	1.76 ± 0.50	1.61 ± 0.45	1.70 ± 0.46	1.38 ± 0.45	1.63 ± 0.43
Task-Driven profile	3.10 ± 0.33	3.05 ± 0.23	3.05 ±0.31	2.96 ± 0.30	3.05 ± 0.28
Utilitarian-Empowering profile	4.00 ± 0.19	3.98 ± 0.18	3.99 ±0.21	3.94 ± 0.27	3.96 ± 0.21
Harmonious-Empowering profile	4.90 ± 0.18	4.92 ± 0.15	4.90 ± 0.19	4.92 ± 0.18	4.88 ± 0.20

### 3.5 Univariate analysis of nurse demographics across different profiles of nurse managers' empowering behaviors

The results showed that there was a statistically significant difference in professional titles among the four profiles (*p* < 0.05), while no statistically significant differences were observed in other variables (all *p* > 0.05) (Table 1). Nurses, senior nurses, and nurse-in-charge had the highest proportion in the Profile 3 (51.1%, 54.2%, 39.9%), while associate chief nurses had the highest proportion in the Profile 4 (50%). There was a statistically significant difference in the total scores of vigor, dedication, and absorption among the four profiles (*p* < 0.001) (Table 6).

**Table 6:** Comparison of work engagement and its dimensions across different latent profiles of nurse managers' empowering behaviors ($\bar{x}$ ± s).

Item	Detached-Control profile (N1)	Task-Driven profile (N2)	Utilitarian-Empowering profile (N3)	Harmonious-Empowering profile (N4)	*F*	*P*	Follow-up pairwise comparisons
Vigor	10.19 ± 4.25	12.41 ± 3.04	14.96 ± 3.49	16.78 ± 4.09	34.71	< 0.001	N4>N3>N2>N1
Dedication	9.69 ± 5.08	11.75 ± 5.57	14.81 ± 4.10	17.19 ± 4.43	39.00	< 0.001	N4>N3>N2>N1
Absorption	10.25 ± 5.45	12.61 ± 3.45	15.11 ± 3.87	17.04 ± 4.43	29.25	< 0.001	N4>N3>N2>N1
Work Engagement	30.13 ± 13.99	36.78 ± 8.96	44.88 ± 10.63	51.01 ± 12.13	39.78	< 0.001	N4>N3>N2>N1

## 4 Discussion

### 4.1 Analysis of the profile results of nurse managers' empowering behaviors

Previous studies have predominantly examined nurse managers' empowering behaviors from a holistic perspective, with few employing latent profile analysis to identify their latent profiles, thereby leaving the heterogeneity of such behaviors insufficiently elucidated. The present study remedied this limitation and revealed that, within the context of Chinese nursing management, nurse managers' empowering behaviors can be clearly identified as four distinct profiles: Detached-Control (4%), Task-Driven (19%), Utilitarian-Empowering (45%), and Harmonious-Empowering (32%), indicating that the majority of nurse managers are capable of adopting relatively positive empowering behaviors, which is consistent with previous studies (16). This finding may be attributed to the fact that all hospitals included in this study were tertiary hospitals, which have generally established relatively comprehensive leadership training systems for nurse managers. As a result, nurse managers have developed a profound understanding of the important value of empowerment in enhancing nurses' sense of responsibility, autonomy, critical reflective ability, communication skills, as well as patient care quality and safety, thereby enabling them to acquire and effectively apply positive empowerment approaches (34). However, approximately one-quarter of nurse managers exhibited the Detached-Control or Task-Driven profile, characterized by relatively low levels of empowering behaviors. This phenomenon may be associated with the hospital assessment mechanism. As the individuals directly responsible for the quality of nursing care in their departments, nurse managers tend to adopt a “hands-on” approach rather than delegating authority in order to avoid nursing errors. Moreover, concerns regarding accountability following delegation may lead them to retain decision-making power (35, 36). This may inhibit nurses' creativity and work efficiency (37, 38), and lead to job burnout (39). Based on these findings, we recommend that nurse managers adopt a competency-based graded delegation approach and establish a “fault-tolerant mechanism” to differentiate between reasonable errors and accountable accidents within the scope of delegated authority. Such measures may help alleviate nurse managers' concerns regarding delegation while ensuring the quality of nursing care (36).

The findings also revealed that nurses, senior nurses, and nurse-in-charge had the highest proportion in the Utilitarian-Empowering profile, while associate chief nurses had the highest proportion in the Harmonious-Empowering profile. This result may be attributed to the following reasons. Junior and intermediate-level nurses are in the early stages of their career development, and their interactions with nurse managers tend to focus primarily on specific work tasks (40). Consequently, their perception of empowering behaviors is more concentrated on instrumental support (e.g., supervisory support, performance incentives), making their perception of the Utilitarian-Empowering profile more prominent. In contrast, as senior nurses, associate chief nurses often undertake additional responsibilities such as teaching and quality control (41). They engage in more frequent and in-depth daily interactions with nurse managers, and their professional competence and work experience are often highly recognized by nurse managers. As a result, nurse managers are more inclined to adopt Harmonious-Empowering behaviors characterized by genuine respect, emotional support, and full delegation, enabling associate chief nurses to better perceive this deeper mode of empowerment. Based on the above findings, it is recommended that nurse managers strengthen emotional support for junior and intermediate-level nurses in their daily management practices, thereby promoting a comprehensive perception of positive empowering behaviors among nurses with different professional titles.

### 4.2 A latent profile analysis of the relationship between nurse managers' empowering behaviors and nurses' work engagement

#### 4.2.1 Nurses under “detached-control” leadership demonstrated the lowest level of work engagement

The study revealed that nurses under “detached-control” leadership exhibited the lowest level of work engagement, which aligns with findings from previous studies in this field (42, 43). Detached-control leaders typically do not attempt to reach agreements with nurses, motivate them, establish standards, or provide feedback opportunities. Instead, they exert pressure on subordinates to comply with imposed expectations without supporting individual autonomy or professional agency (44). This contributes to low job satisfaction and reduced work engagement among nurses. Nurses in hospital settings face multiple significant challenges, including excessive workloads, staff shortages, inadequate resources, outdated infrastructure, and the continuous need for technological upgrading (16). To meet these diverse demands, nurses are required to invest considerable physical and psychological resources in their work. When facing work-related challenges, nurses are unable to obtain the key instrumental support resource of “assistance in overcoming difficulties” from their leaders, leading to rapid depletion of their personal resources due to overconsumption. At the same time, the absence of “respect for staff” and “recognition of work achievements” means a lack of emotional support and constructive feedback, preventing them from replenishing psychological energy through their work. This persistent state of “high depletion–zero replenishment” inevitably reduces work engagement, may trigger workplace bullying, and can result in decreased quality of care and lower patient satisfaction (45, 46).

#### 4.2.2 Under the “Task-Driven” leadership profile, nurses' work engagement was significantly higher than under the “Detached-Control” profile. However, it remained at a relatively low level

The results of this study show that the scores for each dimension of this profile are at a moderate level (ranging from 2.96 to 3.10). Among these, the scores for dimensions such as “assisting in overcoming work difficulties,” “enhancing work meaning,” “recognizing work achievements,” and “granting autonomy” are relatively balanced, while the score for the “respecting staff” dimension is relatively low (2.96). This indicates that leaders of this profile perform adequately in terms of instrumental support but have deficiencies in emotional support. This leadership style resembles task-oriented leadership (47); therefore, this study names this profile “task-driven leadership.”

This study found that nurses' work engagement under task-driven leadership was higher than that under detached-control leadership. Although task-driven leaders fail to provide sufficient replenishment in the emotional support dimension, their limited intervention in “assisting in overcoming work difficulties” enables nurses to obtain necessary instrumental support when facing clinical work obstacles. The moderate level of performance in the “recognizing work achievements” dimension means that leaders provide limited positive feedback to nurses upon task completion. The “enhancing work meaning” dimension also helps nurses enhance their sense of professional value to a certain extent. Additionally, leaders grant a certain degree of autonomous decision-making power in the “granting autonomy” dimension. These factors collectively allow nurses to obtain limited replenishment of the personal resources consumed during work, thereby breaking the “high depletion—zero replenishment” vicious cycle characteristic of the detached-control leadership model and resulting in an increase in work engagement.

However, task-driven leadership scores relatively low in the “respecting staff” dimension (2.96). Nursing work itself is characterized by high emotional demands, and nurses have a greater need for trust and respect from their leaders. When “respect” is insufficient, although nurses can complete tasks according to established requirements, their intrinsic motivation is difficult to fully activate. Consequently, nurses' work engagement often remains at a moderate level, making it difficult to surpass higher performance thresholds (48).

#### 4.2.3 Nurses under both the “Utilitarian-Empowering” and “Harmonious-Empowering” leadership profiles exhibited relatively high levels of work engagement

The most insightful finding of this study lies in clarifying the fundamental distinction between the two advanced profiles—the Utilitarian-Empowering profile and the Harmonious-Empowering profile. Although both provide substantial support, recognition, and empowering behaviors, they differ markedly in their underlying psychological motives and resulting effectiveness. A comparison between these two profiles reveals that both perform well in assisting with difficulties, enhancing work meaning, recognizing achievements, and granting autonomy. The key distinction, however, lies in the intent and depth underlying the dimension of “respecting staff.” For the Utilitarian-Empowering profile, respect may lean more toward maintaining superficial harmony to facilitate task completion, with empowering behaviors carrying a transactional overtone akin to “transactional leadership.” Under transactional leadership, nurses may be driven by material rewards and positive feedback from the nurse managers, factors that satisfy their needs for competence development and external recognition, thereby stimulating their intrinsic motivation (49, 50). In an organizational context characterized by sufficient resources and prompt feedback, utilitarian-empowering leadership can create a relatively stable motivational loop, thereby fostering nurses' continuous engagement with work objectives. Yet, this motivation may become fragile when feedback is delayed or communication is lacking, potentially negatively impacting work engagement (51).

By contrast, harmonious-empowering leadership exemplifies a paradigm that achieves both high performance and high satisfaction. This leadership style is distinguished by its excellence in the relational dimension of “respecting staff.” Authentic and high-level respect effectively fulfills nurses' need for belongingness and fosters a psychologically safe environment. Within such a context, leaders' “assistance” and “recognition” are perceived as genuine support rather than transactional exchanges, and “granting autonomy” is interpreted as authentic trust rather than mere empowerment. As a result, the three fundamental psychological needs—autonomy, competence, and relatedness—are simultaneously and fully met. This state optimally activates nurses' intrinsic motivation, leading to profound work engagement (19).

This study employed latent profile analysis to reveal that nurse managers' empowering behaviors are not homogeneous but can be clearly categorized into four distinct patterns: Detached-Control, Task-Driven, Utilitarian-Empowering, and Harmonious-Empowering. More importantly, these four patterns exhibited a stepwise gradient in relation to nurses' work engagement, ranging from the Detached-Control profile (lowest work engagement) to the Task-Driven profile, followed by the Utilitarian-Empowering profile, and ultimately the Harmonious-Empowering profile (highest work engagement). This finding not only confirms the heterogeneous nature of leadership profiles but also clearly elucidates the differential effects of various empowering patterns on nurses' work engagement. This study provides a basis for tiered and targeted management interventions for nursing administrators. First, for head nurses exhibiting Detached-Control or Task-Driven patterns, interventions should focus on transforming management perceptions and providing empowerment skills training to help them understand the importance of empowering behaviors for nurses' work engagement. Second, for head nurses exhibiting the Utilitarian -Empowering pattern, while acknowledging their effectiveness in providing incentives, efforts should be made to guide them toward attending to nurses' intrinsic motivation and to gradually transition toward the Harmonious-Empowering pattern by increasing emotional support. Third, for head nurses exhibiting the Harmonious-Empowering pattern, their role as exemplary models should be fully leveraged to elevate the overall level of management through experience sharing and mentoring. Future research may further explore the antecedents of these four leadership profiles and their differential effects on outcome variables such as nurses' innovative behavior and intention to stay, thereby providing continued theoretical support for the development of more scientific and precise nursing management intervention systems.

## 5 Limitations

This study has several limitations. First, this study is crosssectional, which limits its ability to make causal arguments. Second, due to constraints related to time, resources, and funding, the sample was primarily drawn from nurses working in tertiary hospitals in Shandong Province. Although the sample size met the requirements of the study, the generalizability of the findings may be limited. Future studies could expand the sample size and include nurses from other regions across China to enhance the robustness and representativeness of the conclusions.

## 6 Conclusion

Against the backdrop of the global nursing shortage, this study offers critical insights into how healthcare institutions can enhance the attractiveness and retention of nursing positions. It not only identifies distinct patterns of empowering behaviors among nurse managers but, more importantly, reveals that combining empowerment with a harmonious atmosphere serves as a key mechanism for improving nurses' work engagement. Relying solely on task-oriented “hard” empowerment or relationship-focused “soft” support is unlikely to yield optimal outcomes. In implementing empowerment strategies, equal emphasis must be placed on building relationships and strengthening support systems. Only by achieving the right balance between granting autonomy and providing supportive guidance can nurses' work engagement and professional wellbeing be truly enhanced.

## Data Availability

The original contributions presented in the study are included in the article/supplementary material, further inquiries can be directed to the corresponding author.
